# The Association Between Total and Regional Body Fat and Bone Mineral Content in Young Athletes: A Cross-Sectional Study

**DOI:** 10.3390/healthcare14030380

**Published:** 2026-02-03

**Authors:** Juliane Correa dos Santos, Jean Carlos Parmegiani De Marco, Tiago Rodrigues de Lima, Clair Costa Miranda, Higor Caetano, Adriana Coutinho de Azevedo Guimarães, Andreia Pelegrini

**Affiliations:** Centro de Ciências da Saúde e do Esporte (CEFID), Universidade do Estado de Santa Catarina (UDESC), Florianópolis 88080-350, SC, Brazil; juliane.santos18@edu.udesc.br (J.C.d.S.); jean.carlos@unoesc.edu.br (J.C.P.D.M.); clair.miranda@edu.udesc.br (C.C.M.); caetanohigor73@gmail.com (H.C.); adriana.guimaraes@udesc.br (A.C.d.A.G.); andreia.pelegrini@udesc.br (A.P.)

**Keywords:** athletes, pediatrics, body composition, bone mineral content, health

## Abstract

Background: Excess body fat during growth has been associated with impaired bone development; however, evidence on the influence of total and regional body fat on bone mineral content (BMC) in physically active youth remains limited. Objective: This study aimed to analyze the association between total and regional body fat and BMC in children and adolescent athletes. Methods: This cross-sectional study included 109 children and adolescents aged 9 to 18 years participating in different sports (indoor volleyball, beach volleyball, swimming, track and field, and basketball). Bone mineral content assessed by DXA and normalized by height (BMC/Height) for the total body less head (TBLH), lumbar spine (L1–L4), and femoral neck was considered the dependent variable. Total and regional (android and gynoid) body fat percentages obtained by dual-energy X-ray absorptiometry (DXA) were treated as independent variables. Associations were examined using multivariable linear regression adjusted for biological and training-related covariates. Results: Total body fat (β = −0.014; *p* < 0.05), android fat (β = −0.011; *p* < 0.05), and gynoid fat (β = −0.014; *p* < 0.05) were significantly and inversely associated with lumbar spine BMC/Height. No associations were observed between total, android, or gynoid fat percentage and TBLH or femoral neck BMC/Height (*p* > 0.05). Conclusions: The inverse and site-specific association of total, android, and gynoid fat with lumbar spine BMC/Height highlights the greater susceptibility of this skeletal site to adiposity-related detriments, underscoring the importance of site-specific monitoring of bone mineral content, even among physically active youth.

## 1. Introduction

Bone health development during childhood and adolescence is a dynamic process, as it is estimated that approximately 90% of peak bone mass has been achieved by the end of adolescence [1,2]. Bone mineral accumulation occurs more gradually in childhood and is amplified in adolescence due to sexual maturation, resulting in rapid somatic growth and profound biological changes that strongly influence skeletal development [1,3]. In this context, understanding the determinants of bone accumulation throughout childhood and adolescence is essential for developing strategies capable of enhancing bone health during the growth process [4].

Although genetic factors account for 60–80% of the variability in bone mass, modifiable aspects (e.g., a balanced diet, body composition, and regular physical activity) may also influence this process [4]. In this context, regular sports participation represents an important strategy for promoting bone health, as the mechanical loading generated by muscle contractions and ground reaction forces stimulates osteogenesis, promoting structural adaptations that result in higher BMD and improved bone geometry [5].

Considering these effects, studies have sought to understand the contribution of different sports modalities to bone development in adolescence. However, evidence indicates that bone benefits are dependent on the specific characteristics of each sport, such that sports with higher ground reaction forces, such as volleyball, track and field, and basketball, present greater osteogenic potential, while non-impact sports, such as swimming, impose less mechanical strain on the skeleton [5,6,7]. This suggests that, in addition to the practice of exercise itself, the specific mechanical loads of each modality are determinants for the beneficial osteogenic effect and for greater bone mineralization [8,9].

However, the magnitude of the beneficial impact of sports participation on bone health may not be homogeneous, as it can be attenuated by other biological factors, such as body composition [10,11], which, in the case of athletes, is closely related to the demands required in each sport [12,13]. In general, direct associations are observed between lean soft tissue and indicators of bone health [14], while higher levels of body fat are associated with low-grade chronic inflammation and oxidative stress [15,16], which can have negative effects on the benefits that sports involvement can provide to bone tissue [14].

In addition to total adiposity, regional fat distribution may exert distinct effects on bone metabolism. Compared with gynoid fat, android fat is more metabolically active and is associated with greater chronic inflammation and insulin resistance [17,18], which may lead to more detrimental effects on bone health [19,20]. These findings suggest that not only excess fat, but also its regional distribution (android or gynoid fat), should be considered in investigations of bone health. The need to isolate and analyze the impact of total and regional adiposity becomes crucial in physically active youth, since fat may exert detrimental effects on bone metabolism even under the osteogenic stimulus of sports [14].

Therefore, given the influence of growth on BMC, analyzing height-normalized bone parameters is essential for accurate assessment during adolescence. Although previous studies have investigated the relationship between adiposity and bone health in youth, evidence remains limited regarding how different distributions of body fat (total, android, and gynoid) interfere with bone development, particularly in adolescents engaged in regular sports practice. Examining factors that may facilitate or impair bone mineral accrual in this population allows for the identification of potential deficits in bone formation and may support preventive and interventional strategies aimed at reducing future risks of osteoporosis and fractures. Thus, the present study aims to analyze the association between total and regional body fat and height-normalized BMC in children and adolescent athletes. The present study hypothesized that total and android body fat would be inversely associated with height-normalized BMC at different bone sites. In contrast, gynoid fat would not be present.

## 2. Materials and Methods

### 2.1. Study Design

The cross-sectional data for this study were derived from the longitudinal microproject “Impact of sports participation on bone density and geometry parameters in adolescent athletes from the state of Santa Catarina: a longitudinal study,” conducted with children and adolescents engaged in regular sports practice in the Greater Florianópolis region, Santa Catarina, Brazil, between October 2021 and June 2023. The data analyzed were collected at two different periods: October 2021 and between February and May 2023. The project was approved by the Human Research Ethics Committee of the Universidade do Estado de Santa Catarina (protocol no. 4,649,164). Participation was contingent upon the signing of the Informed Consent Form (ICF) by the parents/guardians and the signing of the Assent Form (AF) by the adolescents themselves. All ethical principles were followed throughout the study, in accordance with the Declaration of Helsinki.

### 2.2. Participants

The study group was composed of children and adolescents engaged in various sports (indoor volleyball, beach volleyball, swimming, track and field, and basketball), aged 9 to 18 years, recruited from public and private training centers located in the cities of São José and Florianópolis, Santa Catarina, Brazil. The adolescents in this study are classified at level 2, with few approaching level 3, according to the training and performance level classifications proposed by McKay et al. [21], who base these classifications on a combination of criteria that integrate the volume and structure of training, the level of competition achieved, and physical performance metrics. Accordingly, eligibility criteria included the following: (1) individuals aged 9 to 18 years; (2) the absence of a previously diagnosed metabolic disorder capable of influencing physical activity participation; (3) engagement in the sport for at least three months; and (4) submission of the signed ICF and AF by the participants and their guardians.

The selection of sports clubs was based on convenience, considering their proximity to the university where data collection was conducted. The sample size of the original longitudinal project was estimated a priori, considering a statistical power of 80% and an alpha error of 5%. Based on data reported by Agostinete et al. [22], a standard deviation of 0.091 g/cm^2^ and a minimum expected change in bone mineral density of 0.064 g/cm^2^ over the follow-up were assumed. Accordingly, a minimum sample of 35 adolescents per group was estimated, with an additional 20% included to account for potential losses, resulting in a planned sample of 42 participants per group (osteogenic, non-osteogenic, and control), totaling a study group of 126 adolescents. However, the present study represents a cross-sectional analysis derived from this broader project and included only adolescents engaged in sports practice. Therefore, the adequacy of the analytical sample for the current objectives was assessed using post hoc power analysis.

### 2.3. Collection Procedures

Before data collection, contact was made with the coaches and managers of the sports clubs to present the macroproject. After club approval, athletes who met the eligibility criteria were invited to participate voluntarily. Assessments were conducted outside school hours at a physical evaluation laboratory of Universidade do Estado de Santa Catarina, located at the Health and Sports Science Center (CEFID) in Florianópolis. To facilitate participation, it was provided between the training centers and the University, as well as for the return trip. To ensure data standardization and reproducibility, all team members underwent prior training. Furthermore, each specific assessment was consistently performed by the same designated evaluator throughout the entire study period to eliminate inter-rater variability for each variable.

### 2.4. Outcome Variables

Total body less head (TBLH) BMC, lumbar spine (L1–L4) BMC, and femoral neck BMC were assessed via dual-energy X-ray absorptiometry (DXA), using total body and regional scans of the spine and hip performed with a Lunar Prodigy Advance densitometer (GE Medical Systems Lunar, Madison, WI, USA). Data were processed using EnCORE software, version 17 (GE Medical Systems Lunar).

Given that body size and sexual maturation significantly influence DXA-derived BMC [23], and that peak bone accrual and peak height velocity occur at different stages during adolescence [24], adjusting BMC for height is recommended to minimize differences related to body size [23]. Thus, in the present study, BMC was normalized by height (cm), measured with a wall-mounted stadiometer (0.1 cm resolution), generating the variable BMC/Height.

To ensure measurement accuracy, manual adjustments were applied to the regions of interest (ROI). All measurements followed the standards established by the International Society for Clinical Densitometry (ISCD) [25]. Quality control was ensured through daily and weekly equipment calibration [26]. Athletes were instructed to wear light clothing and remove accessories that could interfere with the X-ray beam (e.g., earrings, rings, piercings). They were also advised not to undergo the scan in cases of suspected or confirmed pregnancy and to empty their bladder beforehand. At the time of scheduling, athletes were instructed to avoid moderate-to-vigorous physical activity in the 12 h preceding the evaluation, maintain complete fasting (including water intake) for four hours before the scan, and refrain from using diuretic medications. Female participants who were menstruating on the day of the evaluation had their scans rescheduled.

All DXA scans were conducted by the same evaluator. To assess measurement quality, a pilot study was carried out in which two consecutive scans were performed in 30 adolescent basketball players. The precision of the measurements, expressed as the coefficient of variation (CV), was 0.79% for the total body, 0.80% for the lumbar spine, and 0.75% for the femoral neck. Furthermore, the evaluator’s Least Significant Change (LSC) values were 2.22% (lumbar spine) and 4.16% (femoral neck), both of which are below the maximum limits recommended by the ISCD (5.3% and 6.9%, respectively).

### 2.5. Independent Variables

Percentages of total and regional body fat, specifically for the android and gynoid regions, were investigated as exposure variables. Total body fat percentage reflects the proportion of adipose tissue relative to body mass; android fat corresponds to adiposity accumulated in the abdominal region; and gynoid fat represents fat stored in the hip and thigh regions. All measures were expressed as percentages and obtained from whole-body DXA scans.

### 2.6. Covariates

Adolescents completed a questionnaire containing questions about sociodemographic, biological, and sports-related information to characterize the sample and for statistical control purposes. Sociodemographic data included sex (male/female) and age (in full years). Regarding sports participation, adolescents reported their sports modality (indoor volleyball, beach volleyball, track and field, basketball, and swimming), time of practice in the modality (months), weekly training frequency (days per week), and session duration (minutes). Weekly training volume (minutes/week) was calculated by multiplying weekly frequency by session duration.

In addition, lean soft tissue measurements were also collected from whole-body DXA scans, which were measured in kilograms for characterization and statistical control purposes. Finally, somatic maturation was assessed based on peak height velocity (PHV), using the equations proposed by Moore et al. [27]. This method estimates the maturity offset in years, that is, the time remaining until the individual reaches PHV, or the time elapsed since PHV was achieved. The calculation was performed using the following equations:

$$PHV\;offset\;\left(boys\right)=-7.993994+(0.003294\times (Age\times Height))$$

$$PHV\;offset\;\left(girls\right)=-7.709335+(0.004222\times (Age\times Height))$$

### 2.7. Statistical Analysis

Descriptive and association analyses were conducted using IBM SPSS Statistics version 20.0, adopting a significance level of *p* < 0.05. Data were described using descriptive statistics (frequencies, means, and standard deviations). Data normality was assessed using the Kolmogorov–Smirnov test. Comparisons between sexes were performed using the independent samples *t*-test or the Mann–Whitney test, depending on data distribution. Categorical variables were analyzed using the chi-square test.

Multiple linear regression analyses adjusted for covariates were employed to investigate the association between different distributions of body fat (total fat percentage, and android fat percentage, and gynoid fat percentage) and BMC/Height for TBLH, lumbar spine, and femoral neck. The results were expressed as regression coefficients (β) with their corresponding standard errors (SEs), 95% confidence intervals (95% CIs), coefficient of determination (R^2^) and the effect size from Cohen’s f^2^. The results of this study indicated independence of the residuals, confirmed by the Durbin-Watson test values, which remained between 1.5 and 2.5. Multicollinearity was assessed using the Variance Inflation Factor (VIF) and tolerance. The analysis revealed VIF values below 10 and tolerance values above 0.20.

For each dependent variable related to BMC/height (i.e., TBLH, lumbar spine, and femoral neck), three multiple regression models were constructed, in which only the independent variable representing body fat distribution (total, android, or gynoid) was changed. All models were adjusted for sex, PHV, lean soft tissue, sports modality, time of practice in the modality, and weekly training volume. The selection of covariates was based on evidence from the literature recognizing these variables as determinants of bone mineral content during growth, as they influence bone development through hormonal, mechanical, and maturational pathways [4,6,7,28,29]. As a sensitivity analysis, crude regression models including only body fat measures and BMC/height were conducted and compared with the fully adjusted models to assess the robustness of the observed associations (Table S1).

Post hoc power analyses were conducted using G*Power (version 3.1.9.7) to evaluate the adequacy of statistical power for multiple linear regression. Considering a study sample of 109 adolescent athletes, with seven predictors and a significance level of α = 0.05, the estimated statistical power was 82.8% to detect a medium effect size (f^2^ = 0.15). Despite the recognized influence of sex on bone development, analyses were not stratified due to insufficient statistical power, with only 36.7% for girls and 58.5% for boys, to detect a medium effect size when considering a model with six predictors.

## 3. Results

This study initially included 133 participants. Of these, thirteen were excluded for being in the control group, seven for having less than three months of engagement in their respective sports modality, and four due to missing data. Consequently, the final study group consisted of 109 participants. Overall, there was a higher proportion of boys (60.6%), aged 14 to 18 years (64.2%), and a higher involvement in track and field (25.7%) (Table 1). Regarding sex differences, as shown in Table 1, boys had higher stature, body mass, lean soft tissue, and TBLH and femoral neck BMC/Height compared to girls (*p* < 0.05). Conversely, girls presented higher values for PHV, total body fat percentage, and android and gynoid fat compared to boys (*p* < 0.05).

Table 2 presents the associations between different body fat distributions and bone health indicators in adolescents engaged in sports practice. Regardless of how body fat was considered, no associations were observed with TBLH BMC/Height and with BMC/Height of the femoral neck (*p* > 0.05). Conversely, negative associations were identified between BMC/Height of the lumbar spine amd the percentage of total fat (β = −0.014; 95%CI: −0.027; −0.001; *p* < 0.05), android fat (β = −0.011; 95%CI: −0.020; −0.001; *p* < 0.05), and gynoid fat (β = −0.014; 95%CI: −0.025; −0.004; *p* < 0.05), regardless of sex, PHV, lean soft tissue, sport modality, time spent practicing the modality, and weekly training volume.

Based on sensitivity analyses using simple regression models (Table S1), associations were observed between total and gynoid body fat percentage and TBLH and lumbar spine BMC/Height, as well as between all body fat measures and femoral neck BMC/Height. However, these associations did not remain significant after adjustment for relevant covariates, indicating that the findings from the unadjusted models were likely influenced by confounding factors.

## 4. Discussion

The present study aimed to analyze the association between total and regional body fat and BMC/Height in adolescents engaged in regular sports participation. The results indicated an inverse association between total, android, and gynoid body fat with lumbar spine BMC/Height. Additionally, no associations were observed with TBLH BMC/Height or femoral neck BMC/Height in adolescents participating in sports.

The inverse association between body fat percentage and indicators of BMC and BMD in children and adolescents is consistent with the recent literature [30,31,32]. However, in the present study, inverse associations were observed only between body fat indicators and lumbar spine BMC/Height, whereas associations at other skeletal sites have been reported in previous studies [31,32]. For example, in a study involving approximately eight thousand U.S. children and adolescents aged 8 to 18 years, negative associations were observed between body fat percentage and whole-body and lumbar spine BMD [32]. Similarly, in a study of 377 Brazilian adolescents aged 10 to 19 years, inverse correlations were identified between body fat percentage and indicators of BMC and BMD at the TBLH, lumbar spine, and different femoral regions [31]. This heterogeneity across skeletal sites is further emphasized by Suárez et al. [32], who highlight that different skeletal regions may respond differently to the effects of body fat. This statement is reinforced by the findings of López-Peralta et al. [30], who identified negative correlations only with lumbar spine BMD, specifically among adolescents with obesity.

In line with the heterogeneity across skeletal sites reported in previous studies, sensitivity analyses using crude regression models in the present study indicated inverse associations between body fat measures and BMC/Height across different skeletal sites. However, these associations were attenuated and/or no longer significant after adjustment for sex, PHV, lean soft tissue, and training-related variables. This pattern suggests that the relationships observed in unadjusted models were likely influenced by confounding factors, particularly lean soft tissue and maturational aspects, which are strong determinants of bone mineral accrual during adolescence [1,4,14,28]. Notably, the persistence of the association at the lumbar spine after full adjustment reinforces the site-specific vulnerability of this skeletal region to adiposity-related influences, even in physically active youth.

The literature has reported that excess body fat exerts a direct negative effect on bone metabolism in the pediatric population [4]. This impact is primarily mediated by chronic low-grade inflammation and adiposity-induced oxidative stress, which lead to increased bone resorption and inhibition of osteoblast formation [33]. Furthermore, body fat can interfere with the hormonal axis of bone metabolism, impairing the peak bone mass accrual during adolescence [33]. Although the sample of the present study consisted of athletes, it is hypothesized that the observed results suggest that harmful systemic processes related to body fat may persist and be more evident in specific skeletal regions, as demonstrated by the exclusive inverse association with lumbar spine BMC/Height.

It is speculated that the regional vulnerability of the lumbar spine may lie primarily in its bone morphology and metabolic activity. Bone is composed of two main structures: cortical bone, which is more compact and dense and predominant in regions requiring greater mechanical resistance, such as the femoral neck, and trabecular bone, which is more porous and metabolically active and more abundant in regions such as the lumbar spine [2,34]. The lumbar spine is composed predominantly of trabecular bone, which has a significantly larger metabolic exchange surface compared to the cortical bone of the femoral neck [2,34]. This high metabolic turnover rate makes the lumbar spine a more sensitive systemic sensor to hormonal variations, pro-inflammatory cytokines (released by adiposity), and metabolic disturbances, responding more rapidly and markedly (either negatively or positively) to these stimuli [35]. Although biologically plausible, these interpretations remain hypothetical and must be confirmed in longitudinal or intervention studies.

In addition to morphological specificity, the interaction with mechanical loading from sports participation may modulate the effect of adiposity [5]. Although sport modality was controlled for in the statistical models, the lumbar spine receives impact loads and muscle tension differently from the extremities. While the femoral neck is directly benefited by high-impact sports (such as athletics and basketball), the lumbar spine in adolescents may be subjected to axial compression forces or torsion that, combined with excess body mass (fat), may alter the optimal distribution of mechanical stresses [5]. Thus, it is speculated that, for the lumbar spine, the osteogenic benefit of sport may be more easily offset by adiposity-related inflammatory mechanisms, especially if the local mechanical loading pattern is not optimized or if body fat contributes to unfavorable movement patterns.

### 4.1. Limitations and Strengths

This study presents several limitations that should be considered when interpreting the findings. The cross-sectional design prevents causal inference between body fat and bone parameters. Additionally, nutritional and hormonal data were not collected, which could have contributed to a more comprehensive understanding of the factors influencing bone mineralization. The use of questionnaires also constitutes a limitation, as recall biases (e.g., training volume) may occur. The reduced number of participants in some sports prevented stratified analyses by sport type, which could have provided a more detailed understanding of the specific effects of different mechanical loads on bone health. Although PHV is widely used as an indicator of biological maturation, its accuracy may be reduced in very young individuals or in those at more advanced stages of adolescence. Finally, the absence of sex-stratified analyses, justified by insufficient statistical power, limits the understanding of potential sex differences in the impact of body fat on bone health.

Nonetheless, this study stands out for employing DXA to precisely assess BMC and regional adiposity in a sample of adolescent athletes, while controlling crucial variables such as sexual maturation and training volume, which strengthens the finding regarding lumbar spine vulnerability.

### 4.2. Future Directions

The results of the present study identified the lumbar spine as a point of vulnerability to adiposity in adolescent athletes, reinforcing the need for regional monitoring of body composition. These findings may be particularly relevant for health and sport professionals, as they indicate that mere sports participation may be insufficient to fully protect lumbar spine bone health. Thus, it becomes imperative to implement nutritional and metabolic strategies aimed at optimizing body fat levels, even among highly active individuals. In terms of research, future studies (i.e., longitudinal and interventional) are essential to (1) establish causality between regional adiposity and reductions in lumbar spine BMC, and (2) determine whether decreasing body fat is an effective intervention to reverse this negative impact in physically active adolescents.

## 5. Conclusions

This study investigated the association between total and regional body fat and BMC/Height across different skeletal regions in adolescents engaged in regular sports practice. Total, android, and gynoid body fat were inversely associated with lumbar spine BMC/Height, whereas no significant associations were observed for TBLH or femoral neck BMC/Height. These results indicate that, among the skeletal sites evaluated, the lumbar spine appears to be the most sensitive to the adverse effects that body fat can exert.

## Figures and Tables

**Table 1:** Characteristics of adolescent athletes, considering total sample and by sex.

Variables	Total Sample (n = 109)	Male (n = 66)	Female (n = 43)	*p*-Value
	Mean (SD)	Mean (SD)	Mean (SD)	
Age (years)	14.19 (2.12)	14.14 (2.13)	14.28 (2.13)	0.616 ^¥^
PHV (years)	0.80 (1.97)	−0.03 (1.60)	2.07 (1.82)	<0.001 ^¥^
Height (cm)	166.54 (11.69)	169.88 (11.77)	161.40 (9.61)	<0.001 ^¥^
Body mass (kg)	58.97 (13.85)	60.92 (15.00)	55.97 (11.40)	0.068 ^†^
Lean soft tissue (kg)	42.67 (10.74)	46.55 (11.31)	36.70 (6.19)	<0.001 ^†^
Total fat (%)	24.03 (8.82)	20.04 (8.04)	30.14 (6.09)	<0.001 ^¥^
Android fat (%)	19.47 (11.68)	14.67 (10.33)	26.83 (9.69)	<0.001 ^¥^
Gynoid fat (%)	27.42 (11.21)	21.83 (10.29)	35.99 (5.91)	<0.001 ^¥^
BMC/Height–TBLH (g/cm)	11.39 (2.52)	11.82 (2.68)	10.73 (2.13)	0.026 ^†^
BMC/Height–Lumbar spine (g/cm)	0.34 (0.08)	0.33 (0.09)	0.34 (0.07)	0.456 ^†^
BMC/Height–Femoral neck (g/cm)	0.03 (0.01)	0.03 (0.01)	0.03 (0.01)	0.005 ^†^
Training volume (min/week)	601.70 (351.48)	580.76 (318.54)	633.84 (398.60)	0.803 ^¥^
Time of practice (months)	33.50 (31.29)	30.48 (29.80)	38.12 (33.26)	0.290 ^¥^
	**n (%)**	**n (%)**	**n (%)**	***p*-Value**
Age				<0.001
9 to 13 years	39 (35.8)	24 (36.4)	15 (24.9)	
14 to 18 years	70 (64.2)	42 (63.6)	28 (65.1)	
Sport modality				0.875
Indoor volleyball	26 (23.9)	12 (18.2)	14 (32.6)	
Beach volleyball	11 (10.1)	6 (9.1)	5 (11.6)	
Swimming	18 (16.5)	11 (16.7)	7 (16.3)	
Track and field	28 (25.7)	12 (18.2)	16 (37.2)	
Basketball	26 (23.9)	25 (37.9)	1 (2.3)	

^†^: Independent samples *t*-test; ^¥^: Mann–Whitney test; BMC: Bone Mineral Content; TBLH: Whole body excluding head; PHV: peak height velocity; SD: standard deviation; cm: centimeters; kg: kilograms; %: percentage; g/cm: grams per centimeter; min/week: minutes per week.

**Table 2:** Associations of total body and regional (android and gynoid) body fat percentage with BMC/Height of different body regions in adolescents engaged in sports practice.

	**BMC/Height–TBLH ^†^**			
	β (SE)	95% CI	R^2^	Cohen’s f^2^
Body fat (%)–Model 1	−0.071 (0.177)	−0.422; 0.280	0.800	4.00
Android Fat (%)–Model 2	−0.094 (0.124)	−0.340; 0.152	0.801	4.03
Gynoid fat (%)–Model 3	−0.134 (0.143)	−0.418; 0.151	0.802	4.05
	**BMC/Height–Lumbar Spine ^†^**			
	β (SE)	95% CI	R^2^	Cohen’s f^2^
Body fat (%)–Model 1	−0.014 (0.007) *	−0.027; −0.001	0.743	2.89
Android Fat (%)–Model 2	−0.011 (0.005) *	−0.020; −0.001	0.745	2.92
Gynoid fat (%)–Model 3	−0.014 (0.005) *	−0.025; −0.004	0.749	2.98
	**BMC/Height–Femoral Neck ^†^**			
	β (SE)	95% CI	R^2^	Cohen’s f^2^
Body fat (%)–Model 1	0.000 (0.001)	−0.002; 0.001	0.579	1.38
Android Fat (%)–Model 2	−0.001 (0.000)	−0.001; 0.000	0.585	1.41
Gynoid fat (%)–Model 3	−0.001 (0.000)	−0.002; 0.000	0.582	1.39

^†^: Regression coefficients (β) and standard errors (SEs) were multiplied by 10 for display purposes; *: *p* < 0.05; BMC: Bone mineral content; TBLH: total body less head; β: Regression coefficient; SE: standard error; 95% CI: 95% confidence interval. All models were adjusted for sex, PHV, lean soft tissue, sport modality, time spent practicing the modality, and weekly training volume.

## Data Availability

The raw data supporting the conclusions of this article will be made available by the authors on request.

## Supplementary Materials

The following supporting information can be downloaded at: https://www.mdpi.com/article/10.3390/healthcare14030380/s1, Table S1: Sensitivity analysis of the association between total and regional (android and gynoid) body fat percentage with BMI/Height across different skeletal sites in adolescent athletes using simple regression models.

## Abbreviations

The following abbreviations are used in this manuscript:

AF	Assent Form
BMC	bone mineral content
BMC/Height	Bone Mineral Content normalized by height
BMD	bone mineral density
CI	Confidence Interval
CV	Coefficient of Variation
DXA	dual-energy X-ray absorptiometry
ICF	Informed Consent Form
ISCD	International Society for Clinical Densitometry
LSC	least significant change
PHV	Peak Height Velocity
ROI	Regions of Interest
SD	Standard Deviation
SE	Standard Error
TBLH	total body less head
VIF	Variance Inflation Factor
