# Supplementary material for: The Association Between Total and Regional Body Fat and Bone Mineral Content in Young Athletes: A Cross-Sectional Study
Source: Healthcare (Basel). 2026 Feb 3;14(3):380. doi: 10.3390/healthcare14030380 (PMC12896883; doi:10.3390/healthcare14030380)
Supplement: Supplementary file 1 [file healthcare-14-00380-s001.zip › healthcare-4066097-supplementary.pdf]

Supplementary Table 1. Sensitivity analysis of the association between total and regional (android and gynoid) body fat percentage with BMI/Height across different skeletal sites in adolescent athletes using simple regression models.

	BMC/Height – TBLH†			
	**β (SE)**	**95% CI**	**R^2**	**Cohen's f^2**
Body fat (%) – Model 1	-0.770 (0.266)*	-1.298; -0.242	0.073	0.08
Android Fat (%)– Model 2	-0.327 (0.206)	-0.736; 0.082	0.023	0.02
Gynoid fat (%) – Model 3	-0.776 (0.204)*	-1.181; -0.371	0.119	0.14
	**BMC/Height – Lumbar spine†**			
	**β (SE)**	**95% CI**	**R^2**	**Cohen's f^2**
Body fat (%) – Model 1	-0.019 (0.009)*	-0.036; -0.001	0.038	0.04
Android Fat (%)– Model 2	-0.007 (0.007)	-0.020; 0.007	0.009	0.01
Gynoid fat (%) – Model 3	-0.019 (0.007)*	-0.033; -0.005	0.062	0.07
	**BMC/Height – Femoral neck†**			
	**β (SE)**	**95% CI**	**R^2**	**Cohen's f^2**
Body fat (%) – Model 1	-0.002 (0.001)*	-0.003; 0.001	0.082	0.09
Android Fat (%)– Model 2	-0.001 (0.000)*	-0.002; 0.000	0.047	0.05
Gynoid fat (%) – Model 3	-0.002 (0.000)*	-0.003; -0.001	0.133	0.15

†: Regression coefficients (β) and standard errors (SE) were multiplied by 10 for display purposes; *: $p < 0.05$; BMC: Bone mineral content; TBLH: total body less head; β: Regression coefficient; SE: Standard error; 95% CI: 95% confidence interval.
